# Downregulation of KIF2C and TEKT2 is associated with male infertility and testicular carcinoma

**DOI:** 10.18632/aging.203583

**Published:** 2021-09-30

**Authors:** Haiming Cao, Zi Wan, Fei Wang, Ziyin Liu, Xiaofeng Li, Jianquan Hou

**Affiliations:** 1 The Urology Department, The First Affiliated Hospital of Soochow University, Suzhou, Jiangsu, PR China; 2 The Urology Department, The First Affiliated Hospital of Sun Yat-Sen University, Guangzhou, Guangdong, PR China; 3 The Department of Laboratory Medicine, Peking University Shenzhen Hospital, Shenzhen, Guangdong, PR China; 4 The Urology Department, The Second Affiliated Hospital of Bengbu Medical College, Bengbu, Anhui, PR China; 5 Medical Research Center, Sun Yat-Sen Memorial Hospital, Sun Yat-Sen University, Guangzhou, Guangdong, PR China

**Keywords:** infertility, azoospermia, testis cancer, bioinformatics

## Abstract

Background: Genetic factors are important in spermatogenesis and fertility maintenance, and are potentially significant biomarkers for the early detection of infertility. However, further understanding of these biological processes is required.

Methods: In the present study, we sought to identify associated genes by reanalyzing separate studies from Gene Expression Omnibus datasets (GSE45885, GSE45887 and GSE9210) and validation datasets (GSE4797, 145467, 108886, 6872). The differential genes were used the limma package in R language. Gene ontology and Kyoto Encyclopedia of Genes and Genomes pathway enrichment analyses were performed by the clusterprofier package. The protein-protein interaction network was constructed by the STRING database. The interaction between mRNA and TF was predicted by miRWalk web. At last, The Cancer Genome Atlas data were used to identify hub gene expression levels in GEPIA web.

Results: The results showed that 27 shared genes associated with spermatogenesis. We effectively screen out two genes (KIF2C and TEKT2) and both validated by GSE4797, 145467, 108886 and 6872. Among 27 shared genes, KIF2C and TEKT2 both down-regulated in spermatogenesis. The network of TF-miRNA-target gene was established, we found KIF2C-miRNAs (has-miR-3154, 6075, 6760-5p, 1251-5p, 186-sp)-TFs (EP300, SP1) might work in spermatogenesis.

Conclusions: Our study might help to improve our understanding of the mechanisms in spermatogenesis and provide diagnostic biomarkers and therapeutics targets.

## INTRODUCTION

Infertility, a couple's inability to have children after one year of normal sexual intercourse without protection, affects 10-15% of couples [1–4]. From the latest WHO statistics, nearly 50–80 million persons suffer from infertility [5, 6]. A few studies demonstrate that nearly 50% of all cases of infertility occur because of female factors, 20%–30% male factors, and 20%–30% couples [6–8]. Male infertility is a multifaceted and multiphenotypic disease, which affects about 7% of men worldwide [9]. Male infertility is a complex and heterogeneous phenotypic disease, from complete absence of sperm in testis to changes in sperm quality [10, 11]. Genetic factors account for at least 15% of male infertility. The three main causes of male infertility: spermatogenic function defects; ductal obstruction or disorder; hypothalamic–pituitary axis disorder. A patient with azoospermia is at the highest risk of carrying genetic abnormalities.

With the biotechnology’s improving, we can immediately identify expression diversifications at the transcription level, which beneficially contribute to infertile men. A lot of studies concentrated on differently expressed gene (DEG) analysis have found a quantity of possible molecular goals and diagnostic biomarkers for infertility at the transcription level. Agnieszka et al. discovers that genes, for example, GGN, GSG1, ADCY10, and GTSF1L are down regulated in human beings with azoospermia. Ribosomal protein S3 (RPS3) is recognized by Zhang et al., RPS5, RPS16, RPS23 and RPS6 were downregulated in teratozoospermia [12, 13].

As the study of male infertility is still insufficient, our goal is to analyze whether there are rare potential disease-related genes associated with infertility, and provide clinical evidence [14]. In this study, a comprehensive bioinformatics method was used to study the related genes of spermatogenesis.

## MATERIALS AND METHODS

### Fetching testicular tissue microarray data sets from GEO

**Figure 1:**
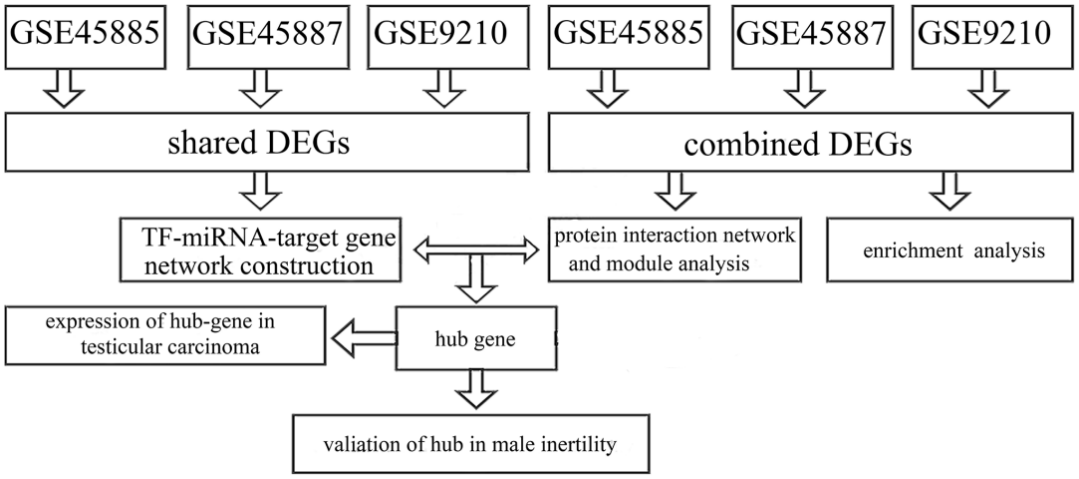
The study procedure.

The data sets were accessed from GEO (http://www.ncbi.nlm.nih.gov/geo/) in the National Center for Biotechnology Information Database (NCBI) utilizing the accession numbers GSE45885, GSE45887, and GSE9210. Data sets GSE45885 was associated by azoospermia and GSE45887 were presented by Agnieszka Malcher and based on the GPL6244 platform ([HuGene-1_0-st] Affymetrix Human Gene 1.0 ST Array [transcript (gene) version, Affymetrix, Inc., USA]). The information of testicular samples in GSE45887 and GSE45885 was accessed from the issued literature (Table 1). The research procedure appeared in Figure 1.

**Table 1:** Characteristics of GEO sample.

**GSE NO.**	**Patients**	**GPL NO.**	**Experiment type**	**Organism**	**Title**	**Description**
GSE45885	31	GPL6244	Expression profiling by array	homo sapiens	Potential biomarkers of non-obstructive azoospermia identified in microarray gene expression analysis	Control group:4; NOA group:27; Age:28–54 (yrs).
GSE45887	20	GPL6244	Expression profiling by array	homo sapiens	The gene expression analysis of paracrine/autocrine factors in patients with spermatogenetic failure compared to normal spermatogenesis	Control group:4; NOA group:16; Age:28–54 (yrs).
GSE9210	58	GPL887	Expression profiling by array	homo sapiens	A testicular gene expression profile for NOA patients, and ART3 as a genetic susceptibility gene for NOA	47 non-obstructive azoospermia (NOA) and 11 obstructive azoospermia (OA) patients
GSE6872	21	GPL570	Expression profiling by array	homo sapiens (semen)	Spermatozoal RNA Profiles	Control group:13; teratozoospermia group:8.
GSE108886	12	GPL10558	Expression profiling by array	homo sapiens (testis)	Spermatogenomics: correlating testicular gene expression to human male infertility	Control group:1; NOA group:8; OA group:3.
GSE145467	20	GPL4133	Expression profiling by array	homo sapiens (testis)	Transcriptome changes in patients with severely impaired spermatogenesis	10 non-obstructive azoospermia (NOA) and 10 obstructive azoospermia (OA) patients
GSE4797	28	GPL2891	Expression profiling by array	homo sapiens	Microarray analysis of human spermatogenic dysfunction	full spermatogenesis (Johnsen Score 10, 12 samples), arrest at the spermatid stage (Johnsen Score 8, 6 samples), arrest at spermatocyte stage (Johnsen Score 5, 5 samples) and Sertoli-cell-only syndrome (Johnsen Score 2, 5 samples).

Abbreviations: NOA: non-obstructive azoospermia; OA: obstructive azoospermia.

We fetched gene expression profile (level 3 information) for Level 3 for Testis carcinoma patients from the TCGA data portal (https://tcga-data.nci.nih.gov/tcga/).

### Microarray data preprocessing

In this study, the original CEL profiles were imported into R (version 3.5, https://www.r-project.org/), and the profiles background was corrected and normalized using the Affy R-package (Bioconductor version 3.6). Affybatch's mas5calls method returns expression sets corresponding to specific genes through multiple probes.

### Differentially expressed gene selection

DEGs were used the limma package (version 3.6). |log2 fold change|>1 and *P* < 0.05 were set at the cutoff values.

### Functional annotation and pathway analysis of DEGs

Go analysis is composed of molecular function (MF) and cellular compartment (CC) and biological process (BP) are usual processes for large-scale genomic data's functional annotation. To learn more the mechanism of DEGs that be involved in infertility, we used clusterprofier R-package to analyze the enrichment of KEGG pathways and GO (version 3.16). In these analyses, *P* < 0.05 was considered statistically significant.

### Protein interaction network and module analyses

The STRING database (http://string-db.org), A protein-protein interaction network that was composed of up-regulated and down-regulated DEGs was built, with a cutoff score more than 0.4. Using the clusterone add-in of Cytoscape v3.6.1 to pick the significant modules from the PPI network (https://cytoscape.org/) with *P* < 0.01 showed statistical importance. The K-core analysis and degree/betweenness/closeness centrality were executed by two add-ins CentiScaPe and Molecular Complex Detection (MCODE) in Cytoscape to illuminate the modules and most significant nodes in the network.

### TF-miRNA-target gene network construction

Interactions between differentially expressed miRNAs and differentially expressed mRNAs and expressed miRNAs were forecast utilizing miRWalk 3.0 (http://mirwalk.umm.uni-heidelberg.de/), and a mark 0.95 was regarded to be the cutoff principle for the estimate analysis in miRWalk. The target mRNAs that was involved in all of these databases were only picked for the further analysis. The interaction between mRNA and TF was predicted by using miRWalk 3.0 and the mark 0.4 was considered as cutoff standard for the estimate analysis in the experimental module of LncBase. After the predicted marks were intersected with DEGs, miRNAs, TFs and mRNAs were picked for further analysis. Cytoscape software (version 3.6.1) was used to visualize the regulatory network.

### Validation and expression of hub-gene in male infertility and testicular carcinoma

At last, the expression levels of hub genes showed in GEPIA (based on TCGA data) (http://gepia.cancer-pku.cn). Next, validation of hub-gene in male infertility by GEO database (GSE4797, 145467, 108886, 6872) (Table 1). *P* < 0.05 was viewed to show a statistically important difference in these analyses.

### Available of data and materials

The datasets analyzed for this study can be found in the GEO datasets (https://www.ncbi.nlm.nih.gov/gds) and TCGA.

## RESULTS

### Analysis of DEGs

**Figure 2:**
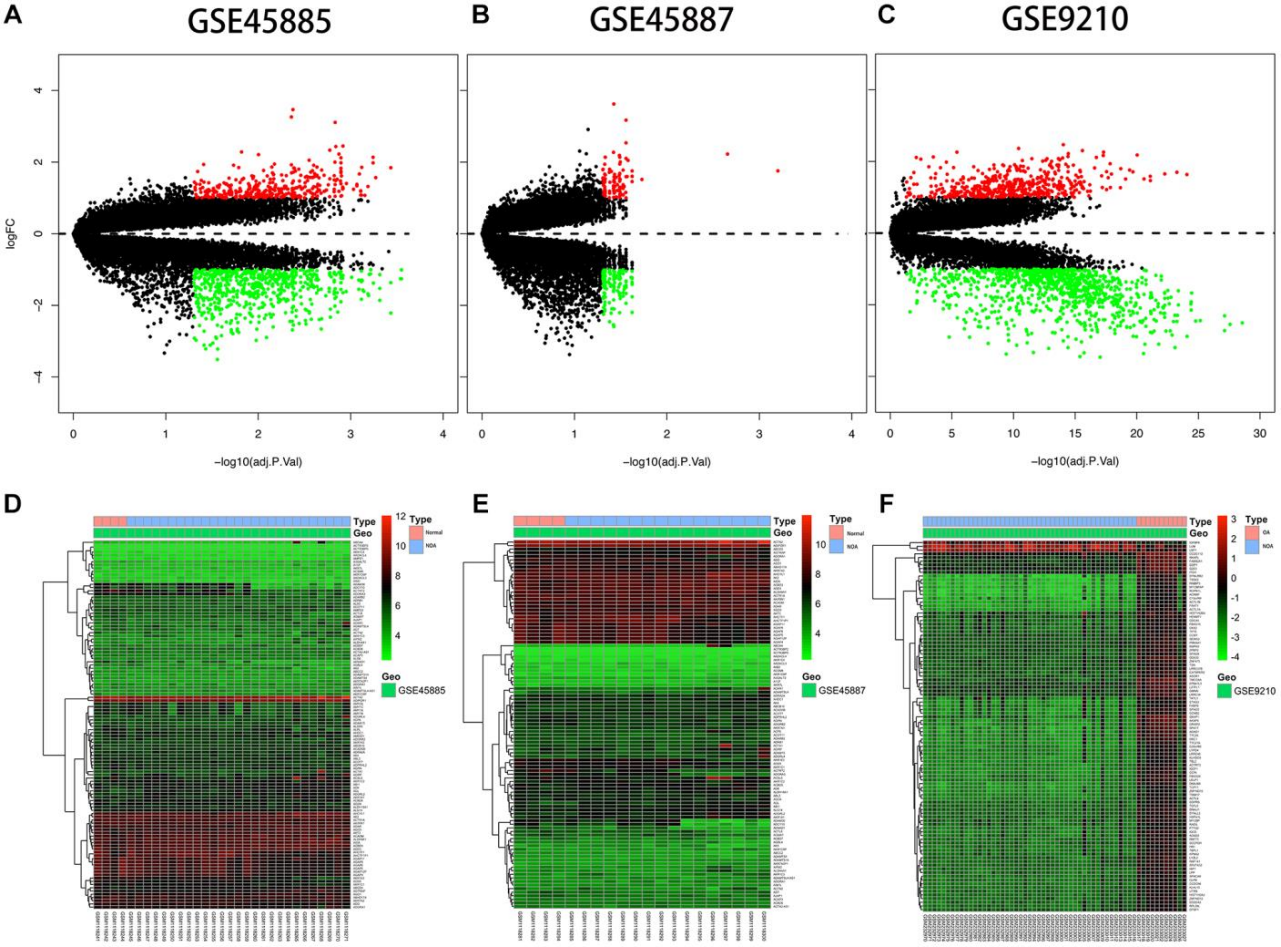
**Differential expression analysis.** (**A**, **B**, **C**) Volcano plot of DEGs. The y-axis is logFC and the x-axis represents -log10 (adjusted *P*-value). The red dots represent the DEGs upregulated and the green dots represent the DEGs downregulated while the black dots represent genes that were not differentially expressed. DEGs, differentially expressed genes; FC, fold change. (**D**, **E**, **F**) Heat map presenting the expression pattern across different samples. The horizontal axis represents sample names. The left vertical axis presented clusters of DEGs, and the top horizontal axis presents clusters of samples. Red represents upregulated genes and green represents downregulated genes.

The expression description information was pre-processed and then analyzed with the Affy package in R language. The entire gene expression was examined. The RNA expression levels are revealed in Figure 2. Differences were revealed by the hierarchical cluster analysis in distribution between normal samples and azoospermic. The consequences showed that grouping was reasonable, and further analysis was undergone by the data with success. Microarray data from the normal samples were compared with those from the azoospermic samples and a sum of 1396 DEGs were discovered.

### Functional annotation and pathway analysis of DEGs

**Figure 3:**
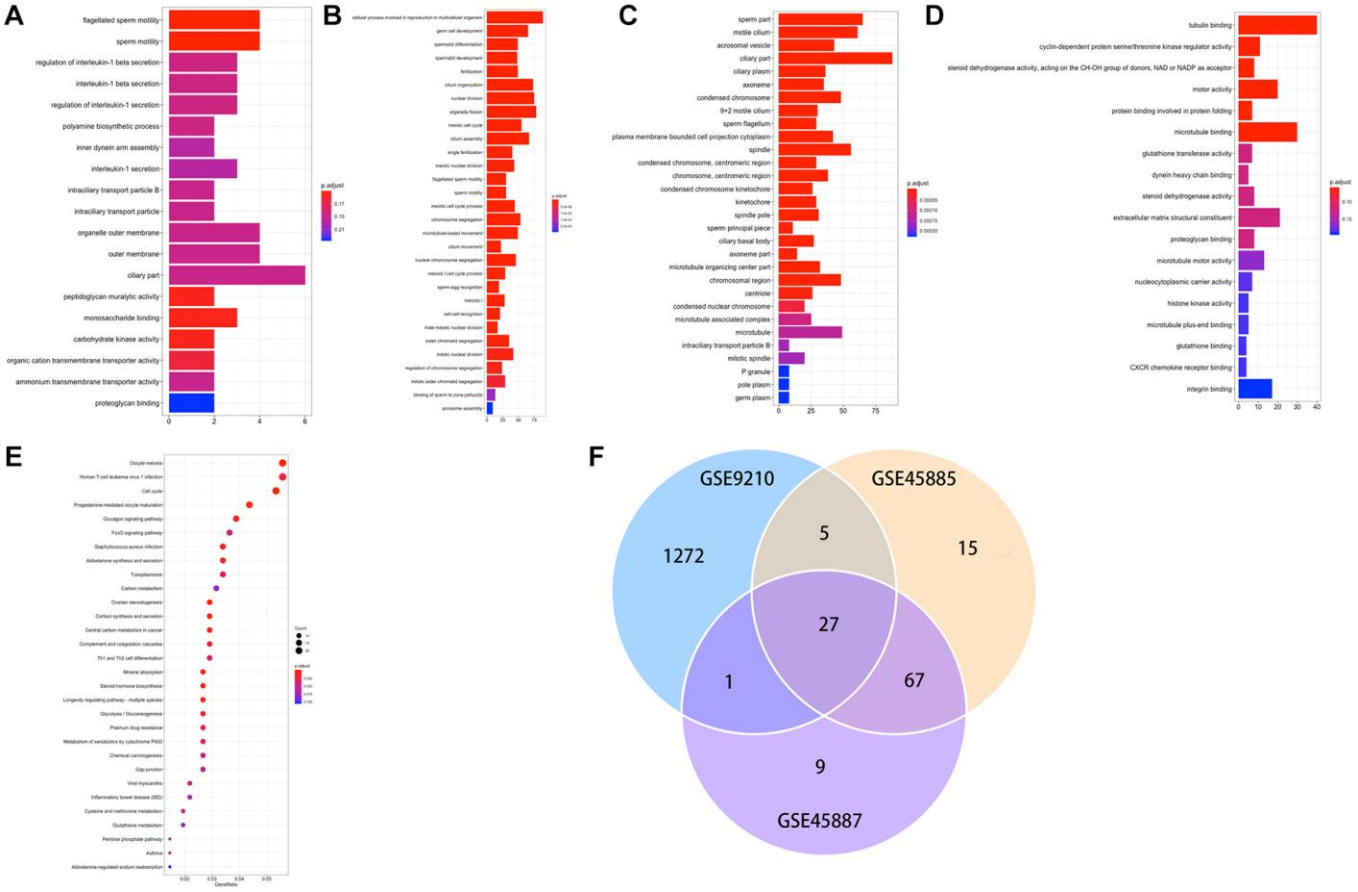
(**A**–**E**) GO and KEGG results of DEGs. GO, Gene Ontology; KEGG, Kyoto Encyclopedia of Genes and Genomes; (**F**) Venn plot of candidate hub genes commonly owned in GSE45885, GSE45887 and GSE9210.

A total of 1396 genes were identified by enrichment analysis, with P (by less than 0.05) statistical significance used to be determined. Figure 3A–3C displayed that GO-MF, CC, BP. The top 10 GO terms that are enriched through up and downregulated genes were primarily enriched in “tubulin binding”, “cyclin-dependent protein serine/threonine kinase regulator activity”, “steroid dehydrogenase activity, acting on the CH-OH group of donors, NAD or NADP as acceptor”, “motor activity”, “protein binding involved in protein folding”, “microtubule binding”, “glutathione transferase activity”, “dynein heavy chain binding”, “steroid dehydrogenase activity”, “extracellular matrix structural constituent” (Figure 3D). The KEGG pathways of DEGs (Figure 3E) were primarily enriched in “Oocyte meiosis”, “Human T-cell leukemia virus 1 infection”, “cell cycle”, “progesterone-mediated oocyte maturation”, “glucagon signaling pathway”, “foxo signaling pathway”, “staphylococcus aureus infection”, “aldosterone synthesis and secretion”, “toxoplasmosis”, “carbon metabolism”. Figure 3F demonstrated 27 DEGs co-expression in three GEO data sets.

### Protein interaction network and module analysis

**Figure 4:**
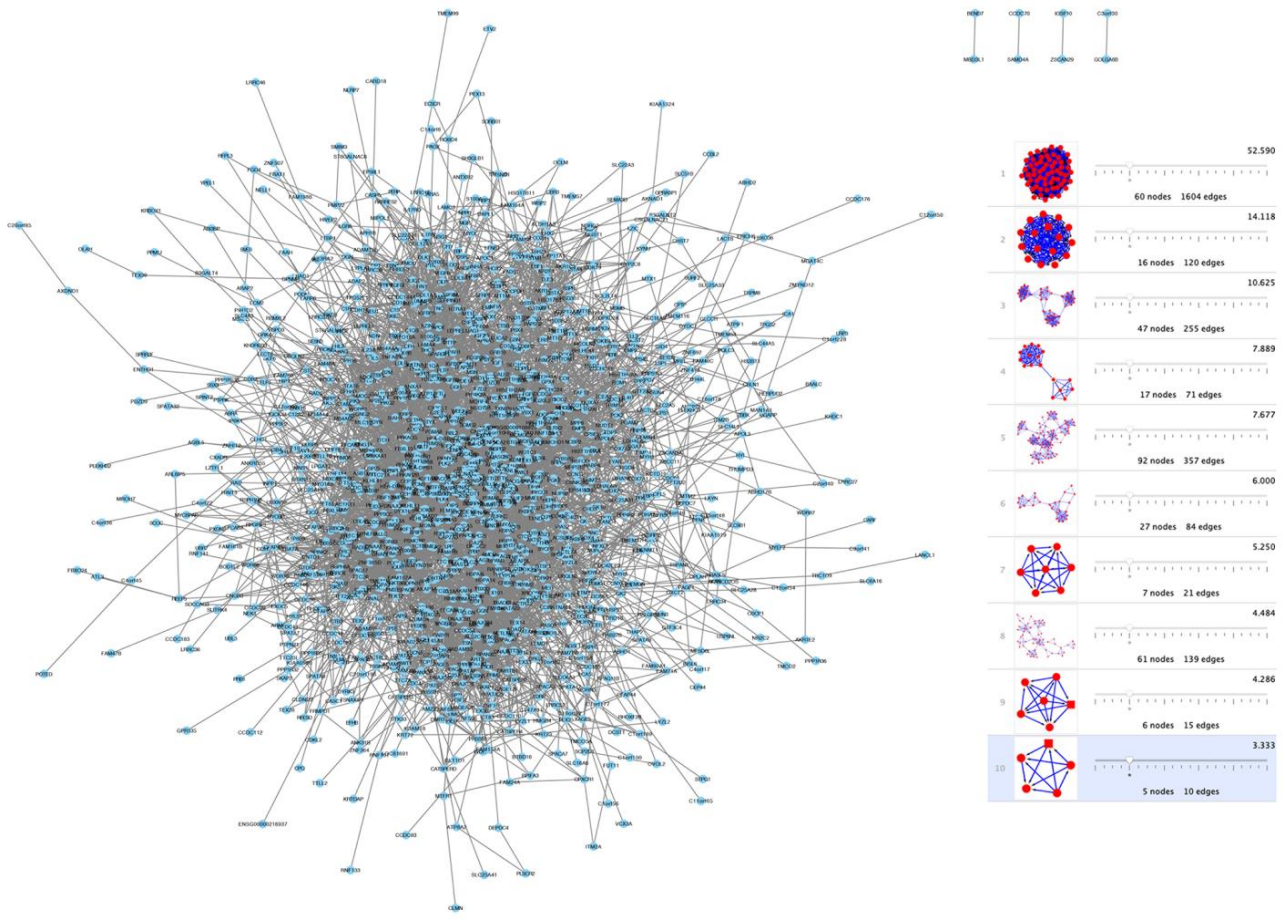
**Network and module analysis of DEGs.** (left) PPI network of DEGs obtained from the STRING database. (right) 10 clusters identified through Cytoscape-MCODE analysis. Abbreviations: DEGs: differentially expressed genes; PPI: protein-protein interaction.

To investigate shared genes' relationship between modules, PPI networks were built by using Cytoscape software that was based on the STRING database. Moreover, the k-core analysis was executed to discover the hub genes and cardinal clusters of PPI networks. By Cytoscape-MCODE analysis, parameters are set as follows: Degree Cutoff: 5, Node Score Cutoff: 0.2, K-Core: 5. 10 clusters of 338 spermatogenesis-associated genes (Figure 4) were identified by us. The hub genes in cluster 1 (Figure 4) that exhibited the highest scores (52.59) were TOP2A, CDT1, KPNA2, TACC3, TEKT2,NUF2, ATAD2, PBK, DLGAP5, TYMS, KIF18A, PTTG1, SPAG5, CDCA8, AURKA, EZH2, CCNB2, EXO1, NCAPG, SMC2, BUB1, KIF15, CDK1, CDC45, ZWILCH, KNTC1, NEK2, KIF20A, MCM4, MAD2L1, CDCA5, CDC20, CCNB1, CENPK, CKS2, OIP5, HMMR, PLK4, ASPM, CDKN3, CEP55, RAD54L, TTK, KIF2C, CENPF, CDCA2, SKA3, SGOL2, RAD51, SPC25, RFC4, MND1, CENPM, CENPU, CASC5, BIRC5, CDC25C, GMNN, RACGAP1, ANLN, UHRF1.

### TF-miRNA-target gene network construction

**Figure 5:**
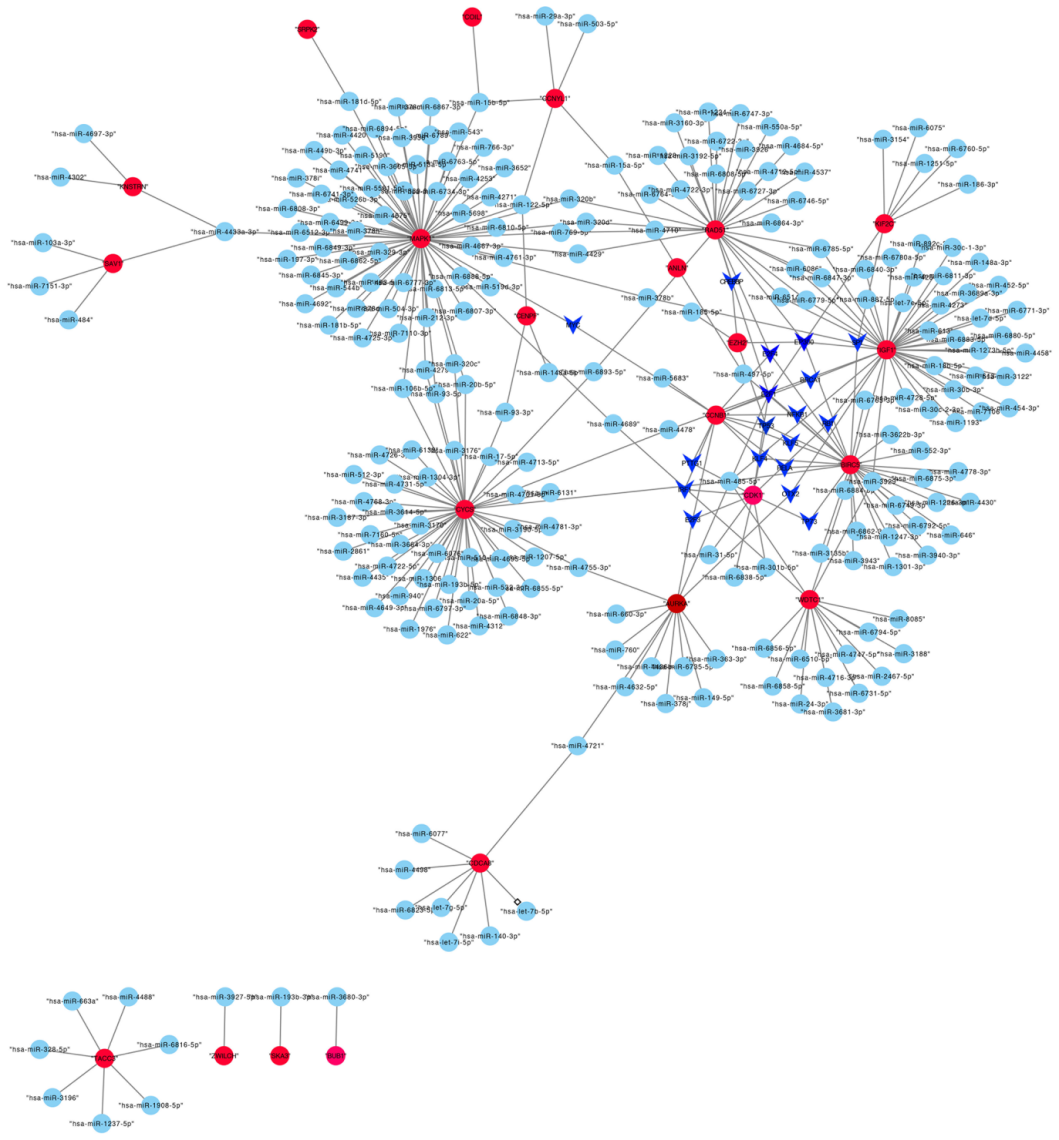
**The transcriptional regulatory network of hub genes, miRNAs, and TFs.** Abbreviations: miRNAs, microRNAs; TFs, transcription factors.

A total of 42 miRNAs could bind to shared genes predicted by miRWalk. Four genes did not have any binding miRNA. The regulatory network of TF-miRNA-target gene was established, involving 18 TFs and 23 hub genes in Figure 5, such as KIF2C-miRNAs (has-miR-3154, 6075, 6760-5p, 1251-5p, 186-sp)-TFs (EP300, SP1).

### Expression of hub-gene in testicular carcinoma

**Figure 6:**
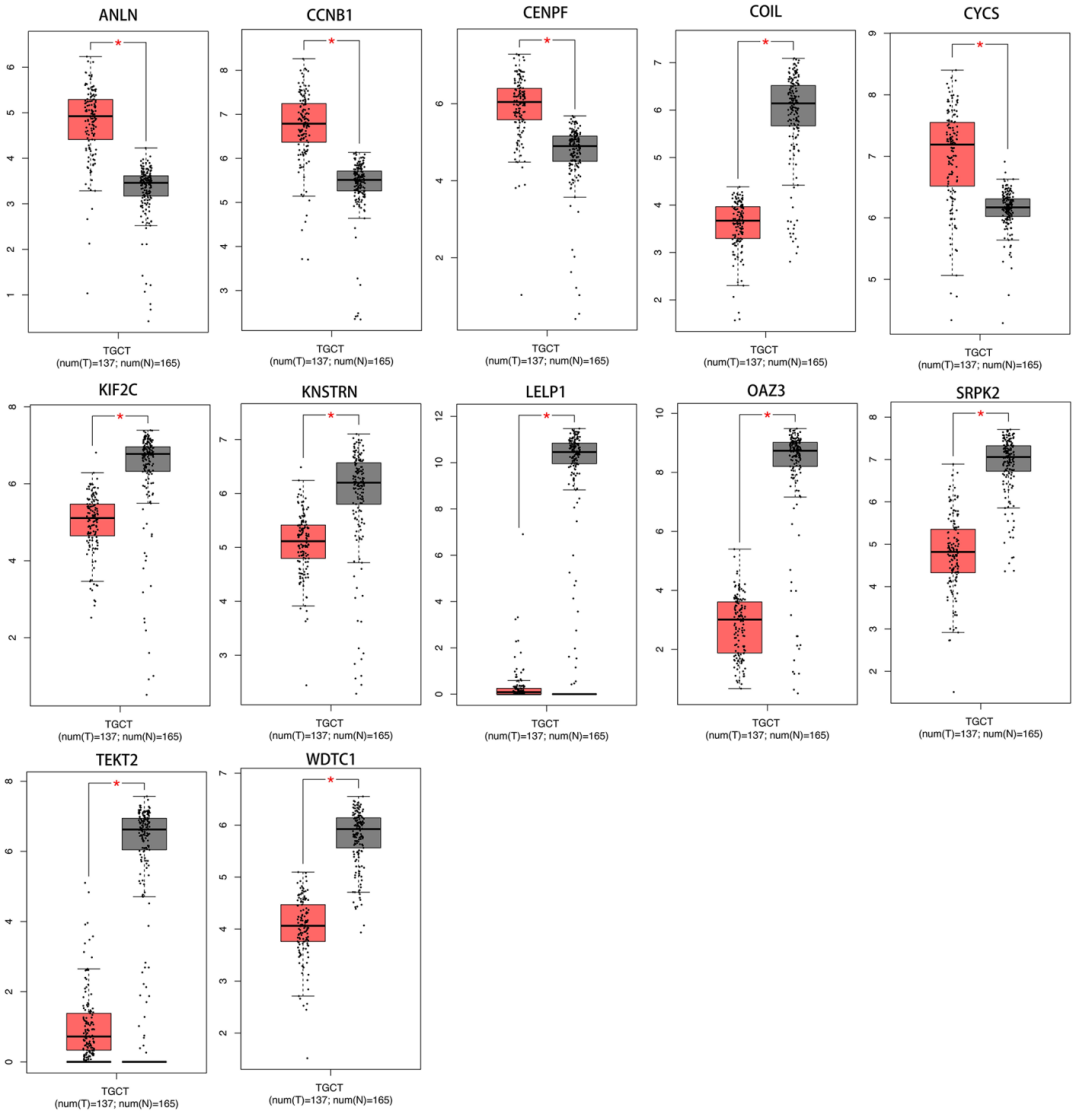
**The transcriptional differences of hub gene levels between colon carcinoma tissues and the para-cancer tissues in TCGA.** TCGA, The Cancer Genome Atlas (^*^p < .001).

A total of 12 genes (ANLN, CCNB1, CENPF, COIL, CYCS, KIF2C, KNSTRN, LELP1, OAZ3, SRPK2, TEKT2, WDTC1) were identified as hub genes. We used TCGA data of testis cancer to validate the hub gene expression with the online tool of GEPIA. All of the hub genes are expressed differently in normal and cancer tissues of testis cancer by the criterion of |logFC|>1 and *p* < .01 (Figure 6).

### Validation of hub-gene in male infertility

**Figure 7:**
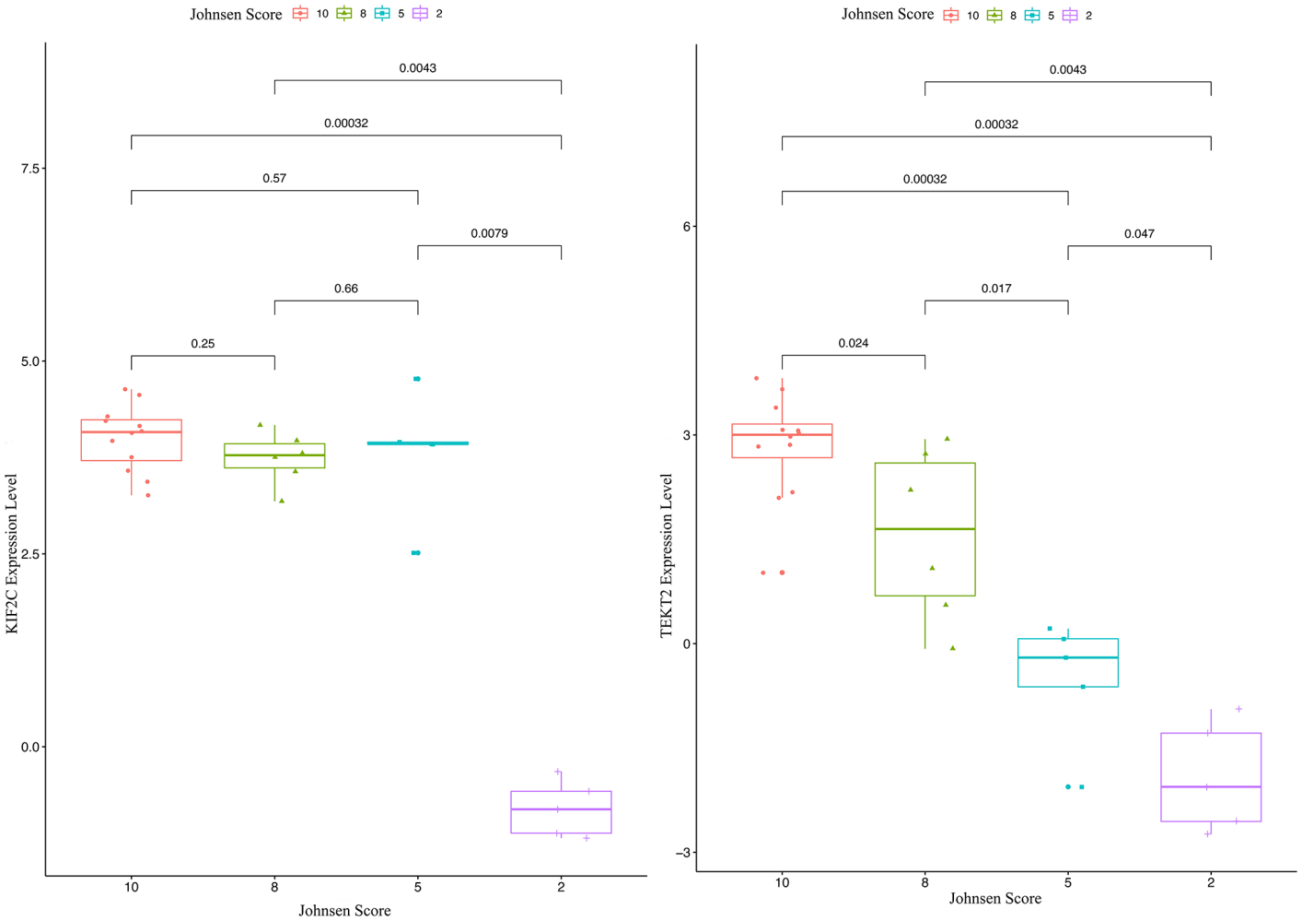
The validation of KIF2C and TEKT2 in GSE4797 associated with male infertility.

**Figure 8:**
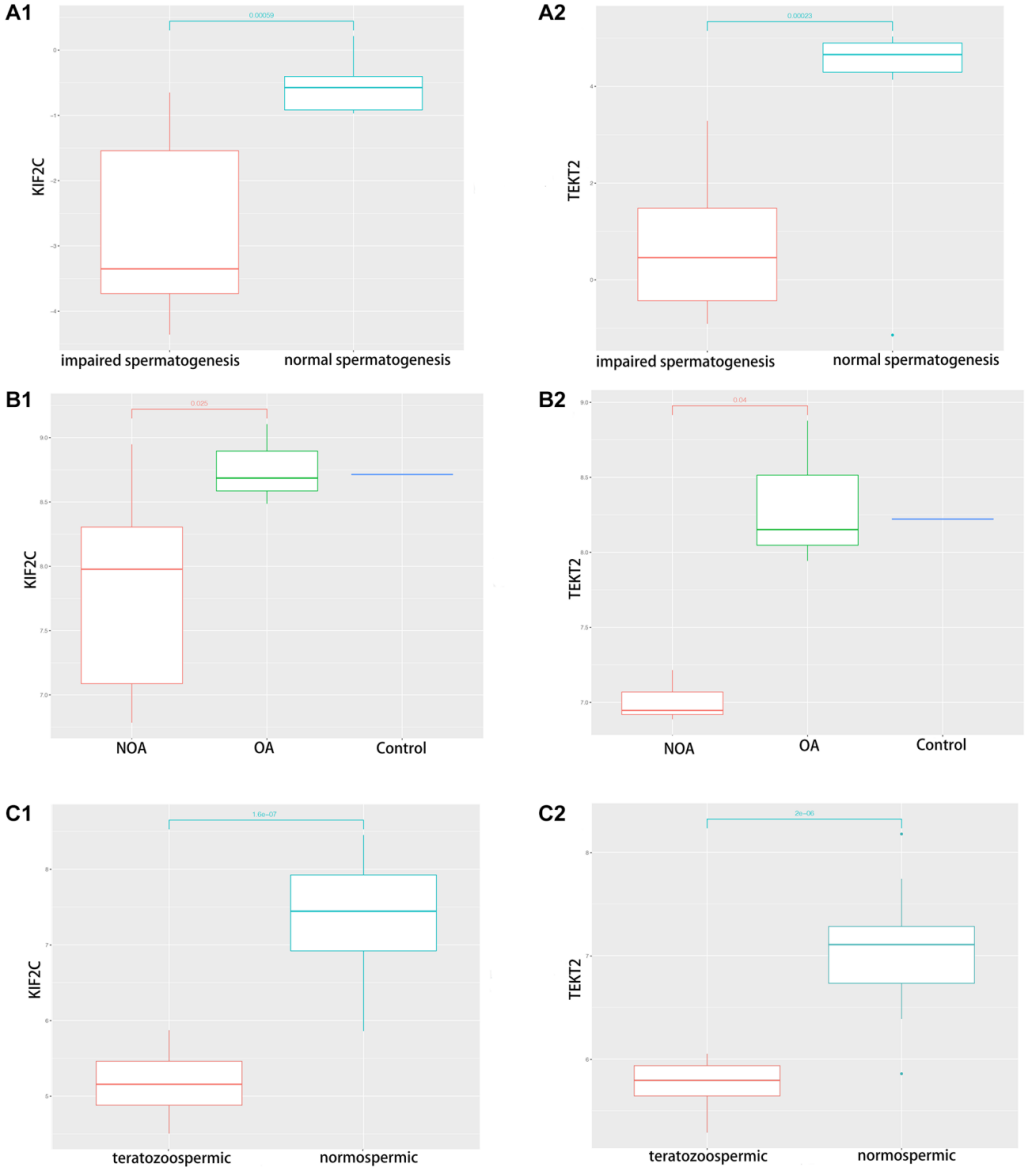
**The validation of KIF2C and TEKT2 in GEO database associated with spermatogenesis.** (**A**) GSE145467; (**B**) GSE108886; (**C**) GSE6872.

Through validation of hub-gene (A total of 12 genes were identified as hub genes in testicular carcinoma) in male infertility by GSE4797 set, we found expression of KIF2C between more than or equal to John score 5 and less than 5 existed significant difference. The expression of TEKT2 decreased with reduction of John score in the Figure 7. The Figure 8 showed the expression of KIF2C and TEKT2 both decreased in three datasets from GEO database.

Furthermore, we found KIF2C might act in infertility and testis cancer through DEG, functional annotation and pathway, protein interaction network and module analysis.

## DISCUSSION

With the development of science and technology, the molecular mechanism of azoospermia has aroused great interest. The study of azoospermia depends on human studies, animal models, organ culture models and cell lines [9, 15]. It has been confirmed that the number of genes and proteins related to male infertility has increased [9, 16]. However, how these biological processes are regulated at the molecular level remains to be elucidated. Therefore, it is necessary to further study the pathogenesis of azoospermia at the molecular level. In this study, we identified the genes associated with spermatogenesis and systematically constructed a comprehensive framework of genes and miRNAs.

In the present study, we sought to identify associated genes by reanalyzing separate studies from GEO datasets (GSE45885, GSE45887, and GSE9210) and validation dataset (GSE4797, 145467, 108886 and 6872) [13, 17, 18]. The results showed that several shared genes associated with spermatogenesis. Finally, we effectively screen out two genes (KIF2C and TEKT2) for validation by GSE4797, 145467, 108886 and 6872. Among these two genes, KIF2C and TEKT2 down-regulated in sperm abnormality. The network of TF-miRNA-target gene was established, we found KIF2C-miRNAs (has-miR-3154, 6075, 6760-5p, 1251-5p, 186-sp)-TFs (EP300, SP1) might work in spermatogenesis. Interestingly, the relative expression levels of KIF2C and TEKT2 had a negative correlation with Johnsen score, which showed potential role of spermatogenesis.

KIF2C (also known as the mitotic centromere-associated kinesin, MACK) is a member of the kinesin-13 family of microtubule (MT)-depolymerizing kinesins, which is critical in the regulation of microtubule dynamics. During cell division, KIF2C inhibits the wrong connection between MT and chromosome [19, 20]. The function of KIF2C in interphase cells is not obvious, although its main localization in nucleus suggests that KIF2C may work in nuclear processes. KIF2C promotes the formation of DNA damage foci, which may involve the migration and aggregation of DSBs (DNA Double Strand Break) [21–25]. We found KIF2C might work in the testis cancer and spermatogenesis.

Tektins (TEKTs), the proteins of the microtubules in Cilia, Flagella, Basal bodies and centrioles [26–28], have been found in various animals, including Filariae, including silk-worms [29], mice [26, 30] and humans [31, 32]. They were originally isolated from sea urchins and are a group of proteins: TEKT-A, -B and -C [33, 34]. On the other hand, five types of TEKTs have been identified in mammals. TEKT2, which is similar to Tektin-t, locates in the main part of human spermatozoa but no immune signal was detected in the middle or at the end of the human sperm. Tektin2, a membrane protein, is responsible for sperm flagellum movement. Previous studies show that CatSper and tektin are associated with male infertility because they play an important role in sperm motility [35]. Tektin2 is essential for the integrity of motilin arm in sperm flagellum. Lack of tektin2 can lead to impaired sperm motility and male infertility [36]. The low expression of tektin2 mRNA was observed in frozen spermatozoa, suggesting that the decrease of sperm motility after cryopreservation may be due to the transcriptional damage of some sperm motility related genes [37].

The miRNAs work in infertility. In 2009, for the first time, expression of miRNAs in a testicular sample of NOA patients compared to fertile control samples evaluated by microarray technology, identified 19 upregulated and 154 downregulated miRNAs [38]. Hsa-miR-141, hsa-miR-429, hsa-miR-7-1- 3p, hsa-miR-34b, hsa-miR-34c-5p, hsa-miR-122 expression levels were different in azoospermia [39, 40]. Through luciferase experiments, miR-525-3p which targets SEMG1 gene and hsa-miR-210 which targets insulin-like growth factor II (IGF2) [41, 42]. The lower expression of hsa-miR-188-3p results in higher expression of MLH1 gene in azoospermia patients and leads to apoptosis in spermatozoa [43].

Functional classification of the miRNA/mRNA pairs using bioinformatics tools indicated that they play a role in spermatogenesis, cell meiosis, cell cycle. We found KIF2C-miRNAs (has-miR-3154, 6075, 6760-5p, 1251-5p, 186-sp)-TFs (EP300, SP1) might work in spermatozoa of infertile men.

Our study also has some limitations. First, more samples could be included in this study and we assessed our results based on published observations. Further *in vitro* and/or *in vivo* experiments would need to be carried out to test reliability of our results. This might reduce the error caused by individual differences of patients.

## CONCLUSIONS

We applied DEG analysis to identify genes associated with azoospermia in this study. Then, through a system biology framework for a comprehensive and systematic biological function- and network-based analysis of azoospermia, we found 27 hub genes and test on the expression of hub-gene in testicular carcinoma (found 12 hub genes were different in testicular carcinoma). Furthermore, we made the validation of hub-gene (A total of 12 genes were identified as hub genes in testicular carcinoma) in male infertility by GSE4797, 145467, 108886 and 6872 and found TEKT2 and KIF2C might work in infertility. The network of TF-miRNA-target gene was established and we found KIF2C-miRNAs (has-miR-3154, 6075, 6760-5p, 1251-5p,186-sp)-TFs (EP300, SP1) might work in spermatozoa of infertile men. Our study might help to improve our understanding of the mechanisms in azoospermia and provide diagnostic biomarkers and therapeutics targets.
